# Strategic environmental assessment—exploring connections between environmental and health issues

**DOI:** 10.1007/s00267-025-02292-1

**Published:** 2025-12-23

**Authors:** Thomas B. Fischer, Ben Cave, Shiu Fung Hung, Tiago Rodrigues, Birgitte Fischer-Bonde, Ainhoa Gonzalez

**Affiliations:** 1 Environmental Assessment and Management Research Centre, Department of Geography and Planning, School of Environmental Sciences, University of Liverpool, Liverpool, UK; 2 Research Unit for Environmental Sciences and Management, Faculty of Natural and Agricultural Sciences, North-West University, Potchefstroom, South Africa; 3 BCA Insight, Dublin, Ireland; 4 School of Geography, University College Dublin, Dublin, Ireland

## Abstract

In this paper, how changes to environmental aspects considered in strategic environmental assessment (SEA) can affect human health is explored, based on a comprehensive and systematic literature review. Whilst health is a key aspect for consideration in SEA, how it should be approached has remained subject to discussion. There is agreement, though, that at a minimum, health impacts from bio-physical environmental changes need be assessed. What this means is elaborated on in this paper. Challenges to and opportunities for an effective consideration of health in SEA are briefly outlined.

## Introduction

Strategic Environmental Assessment (SEA) aims at effectively integrating environmental considerations into policy, plan, programme (PPP), and other strategic decision-making processes. SEA complements Environmental Impact Assessment (EIA) applied to individual projects and aims at establishing the cumulative, synergistic, and long-term impacts of PPPs and other strategies on environmental issues that are either not or only insufficiently considered in project-level assessments (Fischer 2007). By establishing potential negative impacts as well as opportunities for enhancing positive outcomes at a time when concrete development projects are yet to be decided on, SEA can function as a key mechanism for supporting sustainable development, helping to mitigate adverse environmental impacts, whilst pro-actively supporting positive outcomes (Fischer and Gonzalez 2021).

By 2025, SEA had been statutorily implemented in more than 100 countries (González and Fischer 2026). Probably the most widely known SEA regulation internationally is the European SEA Directive from 2001 (EC 2001). In terms of international SEA requirements, the United Nations Economic Commission for Europe SEA Protocol to the Espoo Convention on EIA in a Transboundary Context (UNECE 2003) is also a widely acknowledged and important document. It is of relevance for 56 countries in Europe, Central Asia and North America.

Whilst human health is a key aspect for consideration in SEA (Fischer et al. 2008), there is currently no consensus on how exactly it should be integrated. A starting point for debate can be the most commonly used definition for health from the 1946 constitution of the World Health Organization (WHO):

“Health is a state of complete physical, mental and social well-being and not merely the absence of disease or infirmity” (World Health Organization 2020).

The importance for considering health in SEA is derived from an ability of underlying PPPs and other strategies to affect human health (direct impact) and to influence human behaviour (indirect impact), which can lead to improved health outcomes. Regarding direct impacts, issues such as noise and pollution are relevant. With regards to indirect impacts, as an example, built and natural environments can either encourage or discourage certain activities, such as cycling or walking to work.

It is important that SEA supports alignment with the United Nations 2030 Agenda (United Nations 2015). This consists of 17 Sustainable Development Goals (SDGs) goals and 169 targets. In the context of the SDGs, Menne et al. (2020) noted how health is both an enabler and a major outcome of sustainable development. With regards to health, SDG 3 on good health and well-being, SDG 10 on reduced inequalities, and SDG 11 on sustainable cities and communities are of particular importance. In this context, SEA enables the anticipatory and intersectoral consideration of social and environmental aspects that are interconnected with health, influencing decisions in sectors such as transport, housing, and energy.

A key question of the current debate amongst practitioners and policy makers is to what extent the full range of health determinants (those that are seen to be at the heart of health impact assessments—HIAs; see e.g. Winkler et al. 2021) should also be fully included in SEA. Following WHO (2024), the determinants of health are income and social status, education, the physical environment, social support networks, genetics, health services, and gender. Whilst a wide range of these determinants can potentially be influenced by SEA (Carmichael et al. 2019), some have suggested that the starting point for including health should be the bio-physical environment (UNECE 2022).

It is not the aim of this paper to provide an answer to the question of whether all health determinants should always be considered in SEA. However, even if the focus was on the bio-physical environment only, what aspects should be considered remains unclear, and this is the starting point for this paper. The interlinkages between aspects of the bio-physical environment and human health was a key objective of the ‘Pro Health’ project conducted for the Irish Environmental Protection Agency (EPA) in 2024 to 2025 (https://www.ucd.ie/healthsea/). What follows in this paper is based on the research done for that project.

## Health in SEA: international requirements

The UNECE SEA Protocol (UNECE, 2003) underlines the need for an integration of environmental and health considerations into PPP making processes. Article 1 of the Protocol highlights potential effects of PPPs on human health, alongside other environmental receptors that have been considered traditionally in environmental assessments, including e.g. air, water, and biodiversity. Importantly, the Protocol also calls for an identification of health inequities in particular areas, as well as vulnerable populations on the other. In this context, the Protocol advises that SEA should contribute to equitable outcomes.

The WHO has been instrumental in advancing the consideration of human health within SEA. In this context, the ‘one health’ framework plays a key role for identifying potential health risks associated with ecosystem degradation and zoonotic diseases (WHO, 2023). Besides COVID-19, viruses that have been passed from animals to humans also include others, e.g. Lyme Disease and Ebola. This is of relevance for SEA in the context of e.g. developments that are planned close to natural areas, undisturbed by human activity. Here, SEA should critically assess the possibility of zoonotic disease spread. Also, SEA can advise on pandemic preparedness measures.

In a European context, awareness for the importance of the integration of health into SEAs has gradually increased. A few countries have introduced guidelines and/or recommendations, including the UK (England and Scotland), Georgia, France and Ireland (Roue de Gall et al. 2024; Pyper et al. 2021; Scottish Environment Protection Agency 2019; Roue de Gall et al. 2014; Williams and Fisher 2007).

Subsequently, in this paper, the connections between bio-physical environmental factors to be considered in SEA, according to European Directive 2001/42/EC, and health are explored. Whilst there are other pathways and mechanisms through which human health may be affected by the implementation of PPPs, as indicated above, bio-physical environmental-health interconnections should always be a key concern in SEA. Importantly, as outlined in the introduction, EC and UNECE guidance documents refer to human health without limiting this to environmental factors. Therefore, other health aspects may need to be considered in SEA. However, these are not subject to this paper.

## The need for a proportionate approach

SEA needs to balance the comprehensive assessment of health impacts with the practicalities and limitations of PPP making, as well as with the need to assess a range of environmental aspects. This means that not all aspects will and should always be considered. The International Association for Impact Assessment (IAIA)/European Public Health Association (EUPHA) reference paper for health in Environmental Impact Assessment (EIA) (Cave et al. 2020) has proportionality as one of its principles. Its focus is on the scoping stage and on identifying likely and significant impacts.

**Fig. 1:**
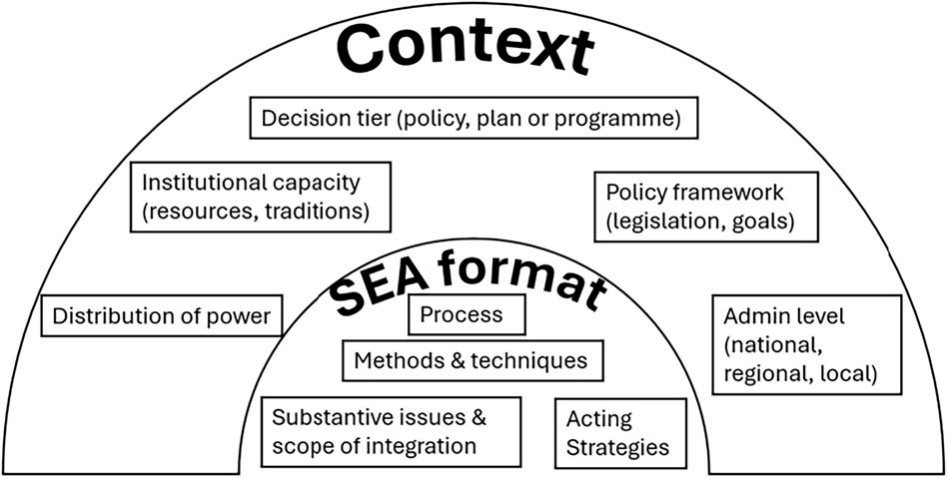
The format of – proportionate – SEA as determined by contextual factors

To assess impacts proportionally, the context of SEA needs to be understood. Whilst the scope of assessment should be set in consultation with relevant authorities, the specific decision tier (i.e. whether it is e.g. a policy, a plan or a programme) and the administrative level (whether the underlying PPP is prepared at e.g. national, regional or local levels) play key roles when deciding on an adequate scope, for example with regards to not repeating data collection processes, assessing the same alternatives or unnecessarily assessing aspects that are not relevant (Fischer 2006; González et al. 2015). With regard to the choice of proportionate impacts, aspects and alternatives, the sector of application is also of importance. Finally, when attempting to devise proportionate SEA, the decision framework within which a particular PPP is prepared and the institutional capacity for conducting SEA (and the distribution of power of different institutional players, which is likely to influence decisions) should be taken into account in order to be able to e.g. counter biases. Figure 1 provides an example for how proportionate SEA (here called ‘SEA format’) can be determined by various contextual factors.

### *Source:* Fischer 2014*, p32*

The principle of proportionality has been included in approaches towards SEA— and also EIA—in a few countries. In France, for example, the 2019 guidance ‘The principle of proportionality in environmental assessment’ recommends that the scope of assessment be adjusted to the relevance of the plan and its expected effects, including on human health. It outlines criteria such as the environmental sensitivity of the area, the nature of planned interventions, and the anticipated impacts, reinforcing the importance of tailoring the assessment to the specific context (Commissariat général au développement durable 2019).

It is important that even if only physical environmental determinants of health were to be considered in SEA, there would still be numerous potential interrelationships that need to be explored. This is why in this paper, health impacts associated with bio-physical environmental issues are elaborated on. This is intended to help those engaging with health in SEA to adequately address the issue.

## Growing concerns on environmental-health interactions

There are currently growing concerns with regards to human health impacts from changes to the bio-physical environment, as well as the health consequences of human impacts on the natural environment. This is associated with various challenges, e.g. the climate change crisis and the recent COVID-19 pandemic. Associated discourses include those surrounding the ‘environmental health’ and ‘one health’ concepts. ‘Environmental health’ focuses on the impact of people on the environment and the impact of the environment on them (Moeller 2005). In 1995, the European Environment Agency defined environmental health as including:

“… both, the direct pathological effects of chemicals, radiation and some biological agents, and the effects (often indirect) on health and well-being (see below) of the broad physical, psychological, social and aesthetic environment, which includes housing, urban development, land use and transport” (European Environment Agency 1995).

Major environmental health risk concerns include exposure to environmental pollutants (e.g. air and noise pollution, heavy metals) and climate-related events (European Environment Agency, 2023). Importantly, the environmental health discourse emphasises the preservation of the natural, living, and occupational (physical and social) environment. Therefore, it is highly relevant for SEA.

Secondly, ‘one health’ is:

“an integrated, unifying approach to balance and optimise the health of people, animals and ecosystems” (World Health Organization 2023).

It follows the thinking around environmental health and emphasises the interconnections of human and animal health, acknowledging that human activities and stressed ecosystems provide opportunities for diseases to transmit to humans and spread (World Health Organization 2023). The thinking surrounding ‘one health’ is said to be key for preventing the next pandemic (European Environment Agency 2020; World Bank 2022).

Zoonotic diseases that are transmitted from animals to humans are viewed as a major threat, and more than 60% of emerging infectious diseases (EIDs) that are reported globally come from animals (World Health Organization 2023). As such, human activities that interfere with wildlife are of fundamental concern. These activities include e.g. agriculture, forestry and urban sprawl (World Bank 2022). SEA plays a key role in assessing impacts in associated PPPs. Therefore, it is important to gather evidence for bio-physical environmental–health interactions, as is done by this paper.

## Literature review methodology

There is a long history of doing research on the relationship between the environment and human health. In this context, a simple desktop search on the Scopus database (January 2024) using the keywords ‘human’ AND ‘health’ AND ‘environment’ returned a total of 78,741 documents. These documents are from various academic disciplines.

The decision to use Scopus for identifying relevant publications for exploring environmental and health interactions was taken due to this being a comprehensive database of peer-reviewed journals in the field of physical and social sciences, with publications from the past several decades being listed. Based on other research done in the field of environmental assessment, Scopus was considered the most adequate of the available options (see e.g. Fischer 2023). There are other databases that were discarded. The Web of Science, for example, is more restrictive than Scopus, in particular with regard to the social sciences and humanities. However, many relevant publications on SEA are from these disciplines. Also, Google Scholar was considered. This probably includes the widest range of academic and non-academic documents. However, anyone can put any document into Google Scholar, which means from an academic point of view, there is a lot of inadequate content. More recently, many AI-generated publications and citations’ manipulations have also been found to have made their way into Google Scholar. Furthermore, Google Scholar is not well organised for keyword searches. Finally, we considered the ‘Newer Dimensions’ database. Whilst this has a greater number of documents than Scopus, they are not freely accessible, as they are behind a paywall (see Singh et al. 2021).

Environmental issues to be considered in SEA are listed in Articles 2 to 7 of the UNECE SEA Protocol and Annex I (f) of the SEA Directive. They include ‘*biodiversity, human health, fauna, flora, soil, water, air, material assets, cultural heritage, including architectural and archaeological heritage, landscape and the interaction among these factors’*. The SEA Directive includes *climatic factors*, and the Protocol refers to *climate*. Furthermore, the SEA Directive includes *population* and the Protocol *natural sites*. The guideline of the EU SEA Directive (European Commission 2004) states that other relevant issues may be included, such as noise.

The aim of the literature search and review was to identify the potential human health impacts associated with environmental issues commonly included in SEA practice. Following the list introduced above, environmental issues to be included in the literature review, therefore, include:

- Air
- Water
- Noise
- Biodiversity/flora and fauna
- Climate
- Soils
- Natural sites
- Landscape/cultural heritage/material assets

As there is a large volume of relevant documents on the environment and human health, restrictions were set to focus the search of literature on the latest and most relevant publications. The search was limited to the documents published within the three years from January 2021 to January 2024, where over 20% of the nearly 80,000 documents from the scopus search mentioned above were published. In cases where there was insufficient information from the articles published in the last three years, the search was extended to five years. For landscape / cultural heritage/material assets, this had to be extended to 10 years. Furthermore, evidence presented by Pyper et al. (2022) is used when explaining and discussing connections.

The literature search considered the subjects of environmental as well as earth and planetary sciences. It was restricted to review articles, resulting in 10,773 potentially relevant documents being identified.

The search string for identifying documents was designed with reference to the environmental issues listed in Articles 2–7 of the UNECE SEA Protocol and Annex I (f) of the SEA Directive, as explained above. Repeated searches were conducted using the keywords of each environmental subject listed, with ‘human’, ‘health’ and ‘review’. The following search string was used:

(KEY (Environmental Subjects *) AND KEY (human) AND KEY (health) AND KEY (review)) AND (LIMIT-TO (SUBJAREA, ‘ENVI’ =) OR LIMIT-TO (SUBJAREA, ‘EART’))

Documents returned from the search were manually screened in order to select those that were relevant in terms of the subjects and scope of the study.

Using the search strings, we found a total of 2136 papers on Scopus. These were manually screened by reading through the title and abstract. We filtered out those papers that focused on subjects that were outside the scope of SEA. For example, studies on indoor environment, occupational health, geographic comparison, and research methods were excluded. After the screening, a total of 238 papers were further reviewed. Amongst those, many did not provide clear statements about environmental and health interlinkages, and some did not present any empirical support for the statements made. Subsequently, we summarise the findings of the 91 papers that provided clear statements about potential environmental and health linkages. References are provided to all 91 papers in the text. Table 1 in the section 'Synthesis' numbers all references used.

## Results

Results of the literature review are summarised with regard to the environmental issues air, water, noise, biodiversity/flora and fauna, climate, natural sites, soils and landscapes/cultural heritage/material assets. The focus of the review is on potential associated health impacts. Implications of the findings for SEA are also briefly explored.

### Air

Air pollution is a significant environmental health risk globally. Using the search string described in the methodology above returned 541 documents that were published since January 2021. After screening and removing documents that did not link environmental issues and health outcomes, 79 documents remained for review. Fifteen of those were found to provide for some evidence on interlinkages.

Fine particulate matter (PM_2.5_, PM_10_), nitrogen dioxide (NO₂) and sulphur (S) are found to be linked to allergies, respiratory diseases, cardiovascular conditions, cancers, kidney malfunctioning, unfavourable pregnancy/birth-related outcomes and premature mortality (Verscheure et al. 2023; Pritchett et al. 2022; Markozannes et al. 2022; Rasking et al. 2022; Song et al. 2023; Sun et al. 2023; Zhu et al. 2024). Furthermore, ammonia (NH_3_), as a substantial contributor of fine particulate matter, is associated with respiratory diseases (Wyer et al. 2022). Importantly, PM_2.5_, PM_10_, ozone (O_3_), NO_2_, and carbon monoxide (CO) were observed to be positively associated with COVID-19 incidences, in which PM_2.5­_ and NO_2_ were correlated with COVID-19 deaths (Chen et al. 2024; Hernandez Carballo et al. 2022). Also, air pollution has been connected with obesity in both children and adults (Zheng et al. 2024).

Air pollution has been observed to be associated with mental health problems. Long term exposure to PM, especially PM2.5, for example, has been correlated with cognitive disorders, ADHD, depression and suicide (Thompson et al. 2023; Liu et al. 2021; Davoudi et al. 2021; Zhang et al. 2022). This is important for consideration in SEA even if it is unlikely that quantification is possible.

Key sources of emissions of particulate matter include traffic and industrial activities, as well as mining. SEA routinely focuses on health impacts of air quality in e.g. urban development plans (including transport) and energy infrastructure plans. A challenge for an effective and consistent inclusion of air quality in SEA is a discrepancy between different air quality standards as the basis for predictions on significance. WHO guidelines (WHO, 2021), for example, have thresholds that are more stringent than EU standards. However, they are not legally binding.

Spatial and land use, as well as sectoral PPPs, e.g. those on industrial development and transport, can either exacerbate or mitigate health risks and their associated SEAs can play key roles in reducing potential negative impacts and enhancing positive outcomes. Air quality is routinely addressed in SEA. However, impacts on the health of affected populations, in terms of e.g. number of people and vulnerable groups at risk of particular illnesses as a consequence of air pollution (both, physical and mental) are only rarely attempted (see Pyper et al. 2022).

### Water

Access to clean water and adequate sanitation is fundamental to public health. Pollution from industrial discharge, agricultural runoff, and urban waste poses significant risks, particularly to marginalised communities. Scopus found 376 documents concerning water quality and human health published between January 2021 and January 2024. After screening and filtering, 34 documents remained for review. Twelve of those were found to provide for some robust evidence on interlinkages.

Drinking water contaminated by chemicals and microbial pathogens is known to pose various health risks. Chemicals such as residue of fertilisers, herbicides and pesticides are identified to increase the risk of developing cancers (El-Nahhal and El-Nahhal 2021; Syafrudin et al., 2021; Picetti et al. 2022; Derbalah and Sakugawa, 2023). Heavy metals in drinking water can damage different internal organs, increasing the risk of developing disease and cancers (Alagan et al. 2023; Zhang et al., 2023). Furthermore, microbial pathogens such as bacteria and parasites can infect a human body’s systems and organs, causing inflammation and chronic diseases (Kristanti et al. 2022; Farrell et al. 2021). Kristanti et al. (2022) also note that there are also concerns that drinking water may play a role in the transmission and outbreak of waterborne diseases.

Antibiotic pollution has been detected in rivers, and antimicrobial-resistant waterborne organisms have been found in coastal and freshwater bodies. This is important, as these have the potential to transmit antimicrobial resistance to humans (Velazquez-Meza et al. 2022; Grenni 2022).

A further concern is plastic pollution in fresh and coastal waters. Microplastics are found to potentially induce gastrointestinal tract inflammation and oxidative stress (Janani et al. 2024). Macro and nano plastics can pass to humans through the food web (Ardestani 2022).

Various aspects of water quality are typically considered in SEA, particularly for those that are prepared for e.g. water management, mining, waste management and transport-related PPPs. In this context, water quality indicators play an important role, including physical (e.g. temperature and turbidity), chemical (e.g. pH value and pesticide concentration), biological (e.g. bacteria and protozoa) and radiological (e.g. uranium) indicators. In SEA, potential health implications, such as the spread of waterborne diseases, are not always included. An example for a framework that supports the incorporation of water-related health outcomes in SEA includes the WHO’s Water, Sanitation, and Hygiene (WASH) initiatives (WHO 2025). These include supporting multi-sectoral technical cooperation and capacity building, provision of data, and integration into disease programmes (e.g. cholera). In the context of SEA, the EU Water Framework Directive (European Parliament 2000) is an important reference for taking water issues into account.

### Noise

Sixty-seven documents published between January 2021 and January 2024 were identified in the Scopus search dealing with noise and human health impacts. After filtering them, nine papers were reviewed. All of them were found to provide for some robust evidence on interlinkages. Among these, traffic noise is the most common type of noise, covered in eight of them.

There is evidence for noise exposure being associated with negative physical as well as mental health and well-being impacts, in particular by causing annoyance (Ata Teneler and Hassoy 2023; Seidler et al. 2023). Elevated noise levels at night time have been associated with sleep disturbance (Smith et al. 2022; Ata Teneler and Hassoy 2023). Exposure to noise was found to be associated with some forms of cognitive impairment, which may impact on reading and language skills (Thompson et al. 2022), potentially adversely affecting children’s learning (Dohmen et al. 2022; Thompson et al. 2022). There are potential associations between noise and other cognitive functions, such as executive functions (which manage behaviours and self-control), but evidence for this to date has remained thin (Thompson et al. 2022). Noise exposure (mainly from traffic) has been found to affect the central nervous system and brain, contributing to an increased risk of neuropsychiatric disorders (Hahad et al. 2022). Road traffic and railway noise have also been found to increase the risks of diabetes mortality (Vienneau et al. 2024). In this context, noise is likely to be potentially associated with obesity (Gui et al. 2022).

Importantly, noise is an example for an environmental issue where effects may not necessarily be negative. For example, there are health benefits associated with e.g. natural sounds. In this context, Zhu et al. (2024) found that exposure to natural sounds (e.g. water or birds) could benefit health recovery and may enhance cognitive performance.

Key sources of noise with potentially negative health impacts include traffic and industrial activities, as well as other sources, particularly connected with urban environments. SEAs have found to underrepresent noise pollution’s health impacts, due to focusing primarily on decibel levels (Pyper et al, 2022). In this context, it is important that perceptions of noise and its disturbance differ in ways that are not captured by decibel measurements. This is connected with e.g. the source of the noise (for example, car traffic is usually perceived to be more disturbing than the noise caused by an approaching and passing train; see UK Health Security Agency, 2023). Furthermore, SEA does not tend to distinguish between affected groups, e.g. vulnerable populations. There are guidelines available that can help addressing shortcomings of current practice. For example, the WHO’s Environmental Noise Guidelines (2018) provide actionable metrics. Non-acoustic factors of importance when considering impacts include e.g. gender, age, education, existing general stress levels, duration of stay at dwelling subjected to noise in the day, window orientation of a bedroom or living room towards the street. In the EU, the Noise Directive is an important reference point for SEA.

### Biodiversity/flora and fauna

For biodiversity/flora and fauna, in the Scopus search, 181 documents were identified that were published between January 2021 and January 2024. After screening and filtering, 24 were reviewed. Six of those were found to provide for some robust evidence on interlinkages.

Biota and ecosystems have been found to affect human health in multiple ways. Natural and urban ecosystems provide services to human societies, including food production (Marselle et al. 2021a; Giglio et al. 2024), as well as cultural, social and scientific values (Marselle et al. 2021a; Giglio et al. 2024). Urban ecosystems, including green and blue infrastructures have been found to affect mental health, wellbeing, and improved social cohesion (Marselle et al. 2021b; Reyes-Riveros et al. 2021, Robinson et al. 2024; Potter et al. 2023). Here, besides the diversity of species, the abundance of specific distinctive species was found to be associated with improved mental health and well-being (Marselle et al. 2021b).

Biodiversity loss is associated with land conversion and extraction of resources. These activities can have substantial implications for human health and well-being. In connection with the ‘one health’ concept introduced earlier, changes in biodiversity can be associated with an increased risk of zoonotic diseases. Furthermore, in association with impacts on biodiversity, ecosystem services can be affected, such as clean air and water quality. Whilst SEA is in a very good position to identify and assess linkages, in practice lack of knowledge of biodiversity and health interactions of those involved in SEA may mean that associated health issues are often not adequately addressed (Pyper et al. 2022).

SEA currently rarely quantifies health impacts of biodiversity loss (Pyper et al. 2022). However, and particularly connected with experiences with the COVID-19 pandemic, there has been an increasing awareness of the ‘one health’ approach, which integrates human, animal, and ecosystem health. This can provide a holistic framework for SEA (Fischer and Cave 2018).

### Climate

Regarding an association between climate and human health, 341 documents were identified by the Scopus search between for the period of January 2021 to January 2024. After screening and filtering for relevance on direct linkages being made between climate change and health, 33 documents were reviewed. Nineteen of those were found to provide for some robust evidence on interlinkages.

An immediate direct impact of climate change is a change in temperatures. Heat is related to ill health, such as dehydration (Faurie et al. 2022). Furthermore, an increase in temperatures is found to elevate the risk of cardiovascular diseases (Faurie et al. 2022; Jurgilevich et al. 2023), kidney diseases (Liu et al. 2021a; Faurie et al. 2022, Jurgilevich et al. 2023), respiratory diseases (Faurie et al. 2022; Z. Zhu et al. 2025), type 2 diabetes and mental disorders (Natur et al. 2022). Heat also increases the morbidity risks of diabetes patients (Moon 2021). Heat has the potential to have adverse effects on pregnant women, increasing the risk of unfavourable birth outcomes (Haghighi et al. 2021; Dalugoda et al. 2022; Syed et al. 2022). Elevated temperatures are found to increase the toxicity of nano-plastics to microorganisms and aquatic animals, but effects on humans remain uncertain (Zhang et al. 2022a).

In addition to heat, extreme weather events and changes in the natural environment are associated with climate change. These also pose multiple health risks to humans. Heatwaves increase the risks of cardiovascular disease, kidney diseases (Jurgilevich et al. 2023), and mental disorders (Li et al. 2023a). Storms and flooding cause accidents and injuries, which can also affect mental health, exacerbating any existing physical and mental health conditions (Anderson et al. 2022; Jurgilevich et al. 2023; Patwary et al. 2024). Wildfires emit particulate matter, which is found to increase the negative effects of non-wildfire particulate matter (Jiao et al. 2024). Respiratory and cardiovascular diseases increase healthcare needs in the aftermath of extreme weather events that lead to, for example, floods, droughts and wildfires (Lee et al. 2023).

Studies found that ocean acidification and warming increase the toxicity of algae blooms and associated harmful effects on humans (Dermawan et al. 2022). Climate change is suggested to affect individual susceptibility to Tuberculosis, particularly among developing countries (Kharwadkar et al. 2022). Among tropical low and middle-income countries, it is also suggested that the transmission rates of several vector-borne diseases may increase (Filho et al. 2022). Children have been found to be more vulnerable to climate-related disease burden (Weeda et al. 2024).

SEA can play a key role for consideration of climate in e.g. national, regional and local spatial and land use PPPs. In this context, it can assess aspects such as the urban heat island effect and suggest mitigation measures. In other sectoral PPPs it can establish potential carbon emissions of different development options and identify how e.g. disadvantaged or vulnerable groups contribute to emissions and whether they are potentially affected to a significantly greater extent than the rest of the population (Fischer et al. 2011; Pyper et al. 2022).

### Soils

The Scopus search resulted in 551 papers covering potential health risks between soils and human health. After filtering for determining direct links between soils and health being made, 14 were reviewed. All of them were found to provide for some robust evidence on interlinkages.

Publications covered the contamination of soils through agricultural activities, mining and the accumulation of microplastics in soil. While contaminated soils from industrial incidences are known to pose significant health risks to humans (as established in e.g. Seveso sites documents; European Parliament 2012), none of the reviewed articles focused on this. It has been suggested that there is a direct connection between soil health and human health, as soil provides multifunctional services to humans, such as food production, nutrient cycling, antibiotics and soil-borne pathogens (Kopittke et al. 2024).

Contaminated soil can affect health directly through ingestion, inhalation and dermal contact. It can also affect human health indirectly, e.g. through consumption of contaminated agricultural products, or water contamination caused by surface runoff (Liu et al., 2023; Nuruzzaman et al., 2025).

Exposure to chemicals used in agricultural activities (e.g. fungicides and pesticides) can cause various illnesses. Pesticides are associated with acute and chronic toxicity, with a potential to cause neurodevelopmental birth defects, asthma, and cancers. There are potential associated long-term health impacts, particularly on children (Kaur et al. 2024). Fungicides (e.g. triazoles) can be toxic and can have adverse effects on the liver, heart, endocrinal, developmental and reproductive systems (Bian et al. 2025).

Mining activities have been associated with heavy metals and potentially toxic trace elements, which are found to pose carcinogenic and non-carcinogenic risks to humans. They affect the kidneys, the liver and skin and include e.g. neuralgia and gastroenteritis. Heavy metals pose higher risks to children than to adults (Hou et al. 2023; Setu and Strezov 2025).

Pharmaceutical contamination is highlighted to be an emerging issue. Pharmaceuticals can enter soil through livestock manure, wastewater, animal excrement and waste disposal (Gentile et al. 2024; Sardar et al. 2025). Pharmaceuticals can also be taken by plants and enter into the food chain. They have been observed to accumulate and can subsequently lead to antibiotic resistance, endocrine disruption, neurological and mutagenic effects (Sardar et al. 2025).

Several papers considered potential health risks associated with microplastics in soils. These are associated with litter, agricultural plastics, sewage sludge, improper sterilisation, plastic mulch films, e-commerce, airborne plastic decomposition, and marine debris (Nath et al. 2024). Microplastics can traverse biological barriers (e.g. intestinal wall and blood-brain barriers) and thus affect vital organs and tissues (Chang et al. 2024). Bioaccumulation of microplastics in the human body can cause inflammation, allergic reaction, oxidative stress, and can induce e.g. respiratory, digestive, urinary, endocrine, nervous, circulation and reproduction issues (Garai et al. 2024; Lee et al. 2024; Shi et al. 2024).

SEA can play a key role in identifying e.g. contaminated areas and sites in spatial and land use PPPs (Fischer 2021). If conducted for new industrial sites, by taking into account potentially produced products, it can identify, early on, mitigation strategies and options for avoiding potential contamination. There are limits to which SEA can take the issues of pharmaceuticals and microplastics into account, though, as these are connected with a range of policy and other decisions that are not associated with spatial and sectoral PPPs that SEA would typically assess.

### Natural sites

The UNECE’s implementation guide of the Aarhus Convention states that “Natural sites may refer to any objects of nature that are of specific value” (Ebbesson et al. 2014, p.51). The health benefits associated with the provision of accessible natural sites have caught much research attention in recent years. The Scopus search identified 52 documents, published between January 2021 and January 2024. After screening and filtering them, 39 were reviewed. Ten of those were found to provide for some robust evidence on interlinkages. Most focused on green spaces, with a few also mentioning blue spaces.

Green and blue spaces provide human health benefits in multiple ways. Generally speaking, they benefit human health with regard to five groups of functions (Zhao et al. 2022; Potter et al. 2023):

- Ecosystem services;
- Absorption of pollutants;
- Spaces for physical activities;
- Alleviation of stress; and
- Enhancement of sleep quality.

Green spaces are found to improve human mental health, can encourage physical activities and result in cardiovascular benefits (Lévesque-Vézina and Lapointe 2024). Green spaces enable an exposure of calming natural environments that can reduce anxiety (Labib et al. 2022) and lower the risk of depression (Liu et al. 2021a; Labib et al. 2022, Bolanis et al. 2024), as well as reducing noise-induced stress (Ferrante et al. 2025). Provision of green spaces has also been found to enhance social cohesion (Huang and Lin 2023) by offering spaces to engage in daily life activities (Huang and Lin 2023). Letting children spend time in green spaces is suggested to help children’s intra- and interpersonal socio-emotional functions and development (Mygind et al. 2021). Exposure to green space has been suggested to have protective effects on non-communicable disease hospital admissions (Luque-García et al. 2022).

SEAs play an important role in helping to avoid or mitigate negative impacts on natural sites and green spaces and enhancing and expanding existing ones (Fischer et al. 2018). This applies to various types of PPPs, including transport, spatial/land use, agricultural, mining, waste, water management and other PPPs. Furthermore, SEA can be applied to development PPPs of natural sites and green spaces.

### Landscape/cultural heritage/material assets

The Scopus search identified 15 documents that dealt with connections of landscape and human health, published between January 2021 and January 2024. No documents were identified with regards to connections between cultural heritage or material assets and health. Due to the low number of documents, the search was extended to include documents published within ten years (i.e. from 2013). With the extended search, 27 documents were identified. Eight of those were found to provide for some robust evidence on interlinkages.

Six documents focused on natural landscapes. There is therefore overlap with natural sites. These state that natural landscapes provide ecosystem services to humans (Coutts and Hahn 2015; Nishi and Hashimoto 2022). Early-childhood exposure to diverse microbiota has also been said to have positive effects on a healthy development (Matthews et al. 2024). Similar to green spaces, exposure to natural landscapes is found to benefit human well-being and to improve mental health, as well as providing spaces for physical activities (Zhang et al. 2021; Hjort et al. 2023; Huang and Lin 2023; Li et al., 2023b). Natural environments have been found to help patients on psychiatric wards to recover (Hjort et al. 2023). In an urban landscape, transportation and the built environment characteristics (e.g. connectivity, aesthetic, safety) affect adults’ motivations for physical activities (Salvo et al. 2018).

The important role SEA can play with regards to landscapes is similar to that identified for natural sites. At times SEA has also been applied to landscape PPPs (Hanusch and Fischer 2011) and development of parks in urban areas can be accompanied by SEA, too.

## Synthesis—Table on environmental-related health risks

Table 1 synthesises findings with regard to the linkages between environmental changes and potential health impacts. These are expressed here as health risks. In addition, vulnerable groups are also listed. Here, 'vulnerable' means more likely to have health impacts/more severe health impacts than the general adult population. Commonly referred to groups in this context include pregnant women and children. The key reason is that the immune system of foetuses, babies and children are underdeveloped, and that the health of mothers is important for the development of them. Another frequently mentioned group is the elderly. The likelihood of exposure is not included in the table. In this context, issues such as poverty or cultural background may be of relevance. Other vulnerable groups include those with pre-existing conditions, including e.g. those with asthma, diabetes or cancer. All environmental changes can potentially affect those with pre-existing conditions. They are therefore not specifically mentioned in the table.

**Table 1:** Environmental related Health risks and vulnerable groups

Environmental aspect	Health risks	Vulnerable groups [not exhaustive]
Air	• Cardiovascular and respiratory disease risks (Markozannes et al. 2022**[1]**; Sun et al. 2023**[2]**; Wyer et al. 2022**[3]**); cancers (Pritchett et al. 2022**[4]**); other diseases and body functions (Rasking et al. 2022**[5]**); viruses (Chen et al. 2024**[6]**) • Allergies (Verscheure et al. 2023**[7]**) • Unfavourable birth outcomes (Markozannes et al., 2022, Song et al. 2023**[8]**, Zhu et al. 2023) **[9]** Mental Health issues (e.g. depression and anxiety, ADHD) (Zhang et al. 2022**[10],**; Davoudi et al. 2021**[11]**, Liu et al. 2021b **[12]**) • Cognitive disorders (Thompson et al. 2023**[13]**) • Development of obesity (Zheng et al. 2024**[14]**) • Deterioration of conditions for those with existing respiratory diseases (Hernandez Carballo et al. 2022**[15]**, Verscheure et al. 2023)	Children, pregnant women, elderly
Water	• Non-communicable disease risk (damage to internal organ and cancer risk) (Janani et al. 2024**[1]**; El-Nahhal and El-Nahhal 2021**[2]**; Syafrudin et al. 2021**[3]**; Picetti et al. 2022**[4]**; Ardestani, 2022**[5]**; Derbalah and Sakugawa 2023**[6]**; Zhang et al. 2023**[7]**) • Microbial infections (Kristanti et al. 2022**[8]**) • Waterborne disease transmissions (Alagan et al. 2023**[9]**) • Antimicrobial resistance (Farrell et al. 2021**[10]**, Grenni 2022**[11]**; Velazquez-Meza et al. 2022**[12]**)	No vulnerable groups specified
Noise	• Sleep disturbance (Smith et al. 2022**[1]**, Ata Teneler and Hassoy 2023**[2]**; Dohmen et al. 2023 **[3]**; Seidler et al. 2023**[4]**; Zhu et al. 2024**[5]**) • Cognitive functions (Thompson et al. 2022**[6]**) • Impact on central nervous system and brain (Hahad et al. 2022**[7]**) • Adiposity (excessive fat issue in the body) (Gui et al. 2022**[8]**) • Diabetes (Vienneau et al. 2024**[9]**)	Children
Biodiversity / flora and fauna	• Changes to immune and digestive system (contamination of food and microbial resistance) (Marselle et al. 2021a **[1]**, Marselle et al. 2021b **[2]**; Potter et al. 2023**[3]**; Robinson et al. 2024**[4]**) • Health issues connected with imbalanced diets (Marselle et al., 2021a, Marselle et al. 2021b, Giglio et al. 2^2024^**[5]**) • Mental health and well-being (Marselle et al. 2021a, Marselle et al. 2021b; Reyes Riveros et al. 2021**[6]**; Potter et al. 2023; Giglio et al. 2024; Robinson et al. 2024)	No vulnerable groups specified
Climate	• Non-communicable disease risks associated with heat (e.g. respiratory, cardiovascular and kidney disease; waterborne diseases) (Liu et al. 2021**[1]**, Faurie et al. 2022**[2]**; Zhang et al. 2022**[3]**; Jurgilevich et al. 2023**[4]**; Zhu et al. 2025**[5]**) • Gastrointestinal problems (Dermawan et al. 2022**[6]**; • Increases in communicable diseases (e.g. tuberculosis)(Kharwadkar et al. 2022**[7]**) • Increases in virus, bacteria, parasites’ and other related illnesses (Filho et al. 2022**[8]**) • Unfavourable birth outcomes (Haghighi et al. 2021**[9];** Dalugoda et al. 2022**[10]**; Syed et al. 2022**[11]**) • Injuries, mental health risks and an exacerbation of existing health conditions due to extreme climate events (flooding, heatwaves and wildfires)(Anderson et al. 2022**[12]**, Natur et al. 2022**[13]**; Jurgilevich et al. 2023; Li et al. 2023**[14]**; Jiao et al. 2024**[15]**; Patwary et al. 2024**[16]**; Weeda ert al. 2024**[17]**; Moon, 2021**[18]**) • Impacts on health services (Lee et al. 2023**[19]**)	Pregnant women, children, the elderly, diabetes patients
Soils	• Carcinogenic and non-carcinogenic risks (e.g. liver, heart, skin endocrine, neuralgia, gastroenteritis and reproductive systems; connected with nitrates, toxins, microplastics and others) (Bian et al. 2025**[1]**; Hou et al. 2023**[2]**; Liu et al. 2023**[3]**; Setu and Strezov 2025**[4]**; Chang et al. 2024**[5]**; Garai et al. 2024**[6]**; Lee et al. 2024**[7]**; Nath et al. 2024**[8]**; Kaur et al. 2024**[9]**; Shi et al. 2024**[10]**; Nuruzzaman et al. 2025**[11]**) • Antibiotic resistance endocrine disruption, neurological effects and mutagenic effects (Sardar et al. 2025**[12]**; Gentile et al. 2024**[13]**; Kopittke et al. 2024**[14]**)	Children

Natural sites	Lack of green spaces deprives populations of (Mygind et al, 2021**[1]**; Labib et al. 2022**[2]**, Zhao et al. 2022**[3]**) Luque-Garcia et al. 2022**[4]**; Bolanis et al. 2024**[5]**; Liu et al. 2023**[6]**; Potter et al. 2023**[7; also mentioned under ‘Flora/Fauna’]**; Lévesque-Vézina and Lapointe 2024**[8]**; Huang and Lin 2023**[9]**; Ferrante et al. 2025**[10]**): • mental health benefits • opportunities for physical activities • respiratory and cardiovascular benefits • opportunities to reduce anxiety, stress, risk of depression	Children, the elderly
Landscape	***See natural sites as well as biodiversity/flora and fauna above*** Also, natural landscapes provide ecosystem services to humans (Coutts and Hahn 2015**[1]**; Salvo et al. 2018**[2]**; Zhang et al 2021**[3];** Nishi and Hashimoto 2022**[4]**; Hjort et al. 2023**[5]**; Huang and Lin 2023**[6; also mentioned under ‘Natural Sites’]**; Li et al. 2023**[7]**; Matthews et al. 2024**[8]**)	Children, the elderly

*NB* Bold numbers in square brackets show the references identified in the literature review

## Challenges and opportunities for an effective integration of health in SEA

Despite knowledge of the numerous connections between environmental impacts and health, practical integration of health into SEA has faced important challenges. These include the availability of only limited guidance documents, data issues (gaps, data protection; see e.g. Dallhammer et al. 2019), the existence of disciplinary silos and resource constraints (Pyper et al. 2021). Currently, most SEA guidelines offer very limited detail on how to assess health impacts (Pyper et al. 2022). This usually leaves those conducting SEA uncertain about what approach to adopt, and what indicators and data sources to use. Reliable data for specific health indicators are frequently not readily available or can only be accessed in fragmented ways (Dallhammer et al. 2019). Environmental and health experts and practitioners are often based in different administrations/departments and do not usually work together. This limits opportunities for collaboration. As a result, health sections in SEA may not integrate well with sections on other environmental issues (Fischer et al. 2010). Finally, on many occasions, SEAs are subject to tight budgets and timelines (Fischer et al. 2023). These are all barriers for a comprehensive consideration of health aspects in SEA. We hope that our paper can be the starting point for addressing challenges.

Opportunities for an effective consideration of health in SEA can be associated with approaches introduced by the WHO and the UNECE. The WHO supports those engaged in SEA with various guidelines. These include e.g. ‘Environmental Noise Guidelines for the European Region’ (WHO 2018) and ‘Global Air Quality Guidelines’ (WHO 2021). These documents provide evidence-based thresholds for environmental determinants that influence health outcomes. They are useful resources for an integration of health into SEA.

The UNECE (2022) introduced the DPSEEA (Drivers, Pressures, State, Exposure, Effects, Actions) Framework, which can serve as a tool for tracing environmental changes to health outcomes. In this context, drivers are understood to be socio-economic factors (urbanisation, economic development) that initiate environmental changes. Furthermore, pressures are changes to environmental conditions, which are triggered by drivers. Examples of pressures include air pollution and habitat loss. The state focuses on the altered state of the environment as a consequence of the pressures applied. Typical examples include degraded air and water quality. Explicit linkages with health are made through the consideration of exposure, which describes the interaction of populations with altered environmental conditions. Effects are health outcomes arising from the exposure. Examples for outcomes include respiratory illnesses and cardiovascular conditions. Finally, actions are the interventions that aim at mitigating risks and at enhancing benefits. Examples include changes to PPPs and observed improvements in environmental and health conditions and outcomes.

## Strengths and weaknesses of the methodology underlying the paper

Whilst the methodological approach underlying this paper has various strengths, there are also some potential weaknesses. Both are briefly outlined here. Our review captures the most recent developments in the field of environmental–health interactions and therefore provides a timely summary of current knowledge. Also, by focusing on those environmental aspects covered by the SEA Directive and the SEA Protocol, actionable insights are created for those engaging with SEA in practice. This addresses the gap between academic research and practical application. Importantly, evidence-based recommendations are provided.

On the other hand, focusing on published papers listed in Scopus means that possible insights from the grey literature (e.g. government reports) have not been considered. Also, only English-language publications were included. Any region-specific findings in non-English formats were therefore shunned. Furthermore, methodologically, we filtered the Scopus search with keywords and subject areas. As different journals set their keywords differently, and Scopus tends to assign subject areas based on the journal rather than article content, papers from certain journals may not have appeared, even if they are about environmental health. Finally, the identification of vulnerable groups was generalised from an environmental science rather than a medical science point of view.

## Conclusions

SEA frameworks, such as the European SEA Directive and the UNECE SEA Protocol, explicitly include health as a key aspect for assessment. However, practical implementation is frequently lagging behind intentions. This is even true when health is considered only through the lens of direct bio-physical environmental impacts. In order to address this issue, in this paper, the authors have provided for a systematic and comprehensive literature review on how various bio-physical environmental aspects that are assessed in SEA are interrelated with numerous potential human health impacts.

Bio-physical environmental aspects to be considered according to the SEA Directive and the UNECE SEA Protocol include air, water, flora and fauna (biodiversity), soils, the climate, natural sites, landscapes cultural heritage and material assets. A wide range of potential physical as well as mental health effects are interrelated with these bio-physical environmental aspects. According to the professional scientific literature, these include risks of (microbial) infections, communicable (e.g. bacteria, viruses, fungi or parasites) and non-communicable (e.g. cardiovascular and respiratory, cancers and diabetes) diseases, risks to cognitive functions, unfavourable birth outcomes, and development of obesity. Children, pregnant women, the elderly and those with pre-existing health conditions may be more seriously affected by certain environmental impacts.

The proportionate consideration of health in SEA is important. Proportionality focuses on a comprehensive assessment of potential health impacts, while acknowledging constraints set by the context within which PPP making is happening. Significant health impacts that are of relevance for a particular PPP need to be prioritised. In this context, issues such as the administrative level, the systematic tier, institutional capacity and power relationships that are present in a particular PPP situation need to be considered in order to be able to conduct assessments are both, rigorous and feasible.

A key challenge for a proportionate consideration of health in SEA has been found to be the absence of specific guidelines. The findings presented in this paper should therefore be useful to those engaging with health in SEA.

## Data Availability

No datasets were generated or analysed during the current study.

## References

[CR1] Alagan M, Chandra Kishore S, Perumal S, Manoj D, Raji A, Kumar RS, Almansour AI, Lee YR (2023) Narrative of hazardous chemicals in water: Its potential removal approach and health effects. Chemosphere 10.1016/j.chemosphere.2023.139178 10.1016/j.chemosphere.2023.139178 37302496

[CR2] Anderson GB, Schumacher A, Done JM, Hurrell JW (2022) ‘Projecting the impacts of a changing climate: tropical cyclones and flooding’. Curr Environ Health Rep 244–262 10.1007/s40572-022-00340-0 10.1007/s40572-022-00340-0 35403997

[CR3] Ardestani MM (2022) ‘Microplastics in the environment: their sources, distribution, and dangerous status’. Water, Air, and Soil Pollution 10.1007/s11270-022-05630-9

[CR4] Ata Teneler A, Hassoy H (2023) ‘Health effects of wind turbines: a review of the literature between 2010 and 2020’. Int J Environ Health Res 10.1080/09603123.2021.2010671 10.1080/09603123.2021.2010671 34856842

[CR5] Bian Y, Zhang Y, Zou P, Zhou Y, Feng X, Wang J, (2025) ‘Triazoles in the environment: an update on occurrence, fate, health hazards, and removal techniques. Environ Res 271;121092 10.1016/j.envres.2025.121092 10.1016/j.envres.2025.121092 39954929

[CR6] Bolanis D, Vergunst F, Mavoa S, Schmelefske E, Khoury B, Turecki G, Orri M, Geoffroy MC (2024) ‘Association between greenspace exposure and suicide-related outcomes across the lifespan: a systematic review’. Sci Total Environ 10.1016/j.scitotenv.2023.167451 10.1016/j.scitotenv.2023.167451 37777126

[CR7] Carmichael L, Townshend T, Lock K, Fischer TB, Sweeting D, Petrokofsky C (2019) Urban planning as an enabler of urban health: challenges and good practice in England following the 2012 planning and public health reforms. Land Use Policy 84:154–162. https://www.sciencedirect.com/science/article/pii/S0264837718307361

[CR8] Cave B, Claßen T, Fischer-Bonde B, Humboldt-Dachroeden S, Martín-Olmedo P, Mekel O, Pyper R, Silva F, Viliani F, Xiao Y (2020) Human health: ensuring a high level of protection. International Association for Impact Assessment

[CR9] Chang N, Chen L, Wang N, Cui Q, Qiu T, Zhao S, He H, Zeng Y, Dai W, Duan C, Fang L (2024) ‘Unveiling the impacts of microplastic pollution on soil health: a comprehensive review’. Sci Total Environ 10.1016/j.scitotenv.2024.175643. 10.1016/j.scitotenv.2024.175643 39173746

[CR10] Chen X, Qi L, Li S, Duan X (2024) ‘Long-term NO2 exposure and mortality: a comprehensive meta-analysis’. Environ Pollut 341 10.1016/j.envpol.2023.122971 10.1016/j.envpol.2023.122971 37984474

[CR11] Commissariat général au développement durable (2019) Le principe de proportionnalité dans l’évaluation environnementale. Minist Transit Écol https://www.actu-environnement.com/media/pdf/news-33914-evaluation-environnementale-principe-proportionnalite.pdf.

[CR12] Coutts C, Hahn M (2015) Green infrastructure, ecosystem services, and human health. Int J Environ Res Public Health 12(8):9768–9798. 10.3390/ijerph120809768 26295249 10.3390/ijerph120809768 PMC4555311

[CR13] Dallhammer E, Schuh B, Gaugitsch R, Derszniak-Noirjean M, Unfried M, Fischer TB, Palenberg D (2019) Territorial impact assessment for cross border collaboration, ESPON: https://archive.espon.eu/TIA-CBC

[CR14] Dalugoda Y, Kuppa J, Phung H, Rutherford S, Phung D (2022) ‘Effect of elevated ambient temperature on maternal, foetal, and neonatal outcomes: a scoping review’. Int J Environ Res Public Health MDPI 10.3390/ijerph19031771 10.3390/ijerph19031771 PMC8835067 35162797

[CR15] Davoudi M, Barjasteh-Askari F, Amini H, Lester D, Mahvi AH, Ghavami V, Rezvani Ghalhari M (2021) ‘Association of suicide with short-term exposure to air pollution at different lag times: a systematic review and meta-analysis’. Sci Total Environ 771 10.1016/j.scitotenv.2020.144882 10.1016/j.scitotenv.2020.144882 33736135

[CR16] Derbalah A, Sakugawa H (2023) ‘Trends in glyphosate use with time in Japan, as well as their relation to surface water concentrations and risk assessment’. Water, Air, and Soil Pollution. Institute for Ionics 10.1007/s11270-023-06733-7

[CR17] Dermawan D, Wang YF, You SJ, Jiang JJ, Hsieh YK (2022) ‘Impact of climatic and non-climatic stressors on ocean life and human health: a review’. Sci Total Environ 10.1016/j.scitotenv.2022.153387 10.1016/j.scitotenv.2022.153387 35081412

[CR18] Dohmen M, Braat-Eggen E, Kemperman A, Hornikx M (2023) ‘The effects of noise on cognitive performance and helplessness in childhood: a review’. Int J Environ Res Public Health 10.3390/ijerph20010288 10.3390/ijerph20010288 PMC9819770 36612610

[CR19] Ebbesson J, Gaugitsch H, Jendroska J, Marshall F, Stec S (2014) The Aarhus convention an implementation guide the aarhus Convention: an Implementation Guide, Second Edition, United Nations. New York: United Nations https://unece.org/environment-policy/publications/aarhus-convention-implementation-guide-second-edition

[CR20] El-Nahhal I, El-Nahhal Y (2021) ‘Pesticide residues in drinking water, their potential risk to human health and removal options’. J Environ Manag 10.1016/j.jenvman.2021.113611 10.1016/j.jenvman.2021.113611 34526283

[CR21] European Commission (2004) Guidance on the implementation of Directive 2001/42/EC on the assessment of the effects of certain plans and programmes on the environment. Brussels Eur Commission. https://environment.ec.europa.eu/law-and-governance/environmental-assessments/strategic-environmental-assessment_en

[CR22] European Environment Agency (1995) Environmental health, EEA Glossary https://www.eea.europa.eu/help/glossary/eea-glossary/environmental-health

[CR23] European Environment Agency (2020) Healthy environment, healthy lives: how the environment influences health and well-being in Europe. Luxembourg: Publications Office of the European Union https://data.europa.eu/doi/10.2800/53670

[CR24] European Environment Agency (2023) Environmental Health Impacts. https://www.eea.europa.eu/en/topics/in-depth/environmental-health-impacts

[CR25] European Parliament and Council of the European Union (2000) Directive 2000/60/EC of 23 October 2000 establishing a framework for Community action in the field of water policy. Official J European Union L 327:1–73

[CR26] European Parliament and Council of the European Union (2001) ‘Directive 2001/42/EC of the European Parliament and of the Council of 27 June 2001 on the assessment of the effects of certain plans and programmes on the environment’. Official J Eur Union L 21(7):30–37

[CR27] European Parliament and Council of the European Union (2012) ‘Directive 2012/18/EU of 4 July 2012 on the control of major-accident hazards involving dangerous substances, amending and subsequently repealing Council Directive 96/82/EC’. Official J Eur Union L 24(7):1–37

[CR28] Farrell ML, Joyce A, Duane S, Fitzhenry K, Hooban B, Burke LP, Morris D (2021) ‘Evaluating the potential for exposure to organisms of public health concern in naturally occurring bathing waters in Europe: a scoping review.’, Water Res 206 10.1016/j.watres.2021.117711 10.1016/j.watres.2021.117711 34637971

[CR29] Faurie C, Varghese BM, Liu J, Bi P (2022) ‘Association between high temperature and heatwaves with heat-related illnesses: a systematic review and meta-analysis’,. Sci Total Environ 10.1016/j.scitotenv.2022.158332 10.1016/j.scitotenv.2022.158332 36041616

[CR30] Ferrante M, Rapisarda P, Castrogiovanni M, Filippini T, Oliveri Conti G, Vinceti M (2025) ‘Urban greenness for the protection of adverse effects of noise on human health: a PRISMA systematic review’, Sci Total Environ 10.1016/j.scitotenv.2025.179415 10.1016/j.scitotenv.2025.179415 40245511

[CR31] Filho WL, Ternova L, Parasnis SA, Kovaleva M, Nagy GJ (2022) ‘Climate change and zoonoses: a review of concepts, definitions, and bibliometrics.’, Int J Environ Res Public Health 10.3390/ijerph19020893 10.3390/ijerph19020893 PMC8776135 35055715

[CR32] Fischer TB (2006) Strategic environmental assessment and transport planning: towards a generic framework for evaluating practice and developing guidance. Impact Assess Proj Appraisal 24(3):183–197. 10.3152/147154606781765183

[CR33] Fischer TB (2007) Theory and practice of strategic environmental assessment – towards a more systematic approach. London: Earthscan

[CR34] Fischer TB (2014) Health and Strategic Environmental Assessment. In: Fehr R, Martuzzi M, Nowacki J, Viliani F (eds) Health in Impact Assessments, WHO, EUPHA and IAIA: pp 23–46 http://www.euro.who.int/en/health-topics/environment-and-health/health-impact-assessment/publications/2014/health-in-impact-assessments-opportunities-not-to-be-missed

[CR35] Fischer TB (2023) Impact assessment publishing–observations and reflections after 7 years of being editor of impact assessment and project appraisal. Impact Assess Proj Appraisal 41(3):175–180. 10.1080/14615517.2023.2207269

[CR36] Fischer TB (2021) A review of impact assessments related to remediation and further re-use of contaminated sites. In: WHO/EURO:2021-2187-41942-57585 (Urban redevelopment of contaminated sites: a review of scientific evidence and practical knowledge on environmental and health issues. pp 143–165. https://apps.who.int/iris/bitstream/handle/10665/340944/WHO-EURO-2021-2187-41942-57585-eng.pdf

[CR37] Fischer TB Cave B (2018) ‘Health in impact assessments–introduction to a special issue’. Taylor and Francis Ltd., pp. 1–4 10.1080/14615517.2017.1363976

[CR38] Fischer TB, Gazzola P, Jha-Thakur U, Belcakova I, Aschemann R (eds) (2008) Environmental Assessment Lecturers’ Handbook, ROAD Bratislava.

[CR39] Fischer TB, Fonseca A, Geißler G, Jha-Thakur U, Retief F, Alberts R, Jiricka-Pürrer A (2023) Simplification of environmental and other impact assessments–results from an international online survey. Impact Assess Proj Appraisal 41(3):181–189. 10.1080/14615517.2023.2198839

[CR40] Fischer TB, González A (2021) ‘Conclusions - Towards a Theory of Strategic Environmental Assessment?’. In: Fischer TB, González A (eds) Handbook on strategic environmental assessment. Cheltenham, U.K.: Edward Elgar Publishing Ltd., pp. 425–437 10.4337/9781789909937.00018

[CR41] Fischer TB, Jha-Thakur U, Fawcett P, Nowacki J, Clement S, Hayes S (2018) Consideration of urban green space in impact assessment for health. Impact Assess Proj Appraisal 36(1):32–44. https://www.tandfonline.com/doi/pdf/10.1080/14615517.2017.1364021

[CR42] Fischer TB, Martuzzi M, Nowacki J (2010) The consideration of health in strategic environmental assessment (SEA). Environ Impact Assess Rev 30(3):200–210. 10.1016/j.eiar.2009.10.005

[CR43] Fischer TB, Potter K, Donaldson S, Scott T (2011) Municipal waste management strategies, strategic environmental assessment and the consideration of climate change in England. J Environ Assess Policy Manag 13(4):541–565. 10.1142/S1464333211004000

[CR44] Garai S, Bhattacharjee C, Sarkar S, Moulick D, Dey S, Jana S, Dhar A, Roy A, Mondal K, Mondal M, Mukherjee S, Ghosh S, Singh P, Ramteke P, Manna D, Hazra S, Malakar P, Banerjee, H., Brahmachari, K. and Hossain, A. (2024) ‘Microplastics in the soil–water–food nexus: inclusive insight into global research findings’, Sci Total Environ 10.1016/j.scitotenv.2024.173891 10.1016/j.scitotenv.2024.173891 38885699

[CR45] Gentile A, Di Stasio L, Oliva G, Vigliotta G, Cicatelli A, Guarino F, Nissim WG, Labra M, Castiglione S (2024) ‘Antibiotic resistance in urban soils: dynamics and mitigation strategies’. Environ Res 10.1016/j.envres.2024.120120 10.1016/j.envres.2024.120120 39384008

[CR46] Giglio VJ, Aued AW, Cordeiro CAMM, Eggertsen L, S. Ferrari D, Gonçalves LR, Hanazaki N, Luiz OJ, Luza AL, Mendes TC, Pinheiro HT, Segal B, Waechter LS, Bender MG (2024) A global systematic literature review of ecosystem services in reef environments. Environ Manag 73(3):634–645. 10.1007/s00267-023-01912-y 10.1007/s00267-023-01912-y 38006452

[CR47] González A, Fischer TB (2026) Strategic environmental assessment. In: Fischer, TB, Noble B, Retief F, Jha-Thakur U, Bice S, Montano M, (eds) Encyclopaedia of Impact Assessment, Edward Elgar, Cheltenham

[CR48] González A, Thérivel R, Fry J, Foley W (2015) Advancing practice relating to SEA alternatives. Environ Impact Assess Rev 53:52–63.

[CR49] Grenni P (2022) Antimicrobial resistance in rivers: a review of the genes detected and new challenges. Environ Toxicol Chem 41(3):687–714. 10.1002/etc.5289 35191071 10.1002/etc.5289

[CR50] Gui SY, Wu KJ, Sun Y, Chen YN, Liang HR, Liu W, Lu Y, Hu CY (2022) Traffic noise and adiposity: a systematic review and meta-analysis of epidemiological studies. Environ Sci Pollut Res 29(37):55707–55727. 10.1007/s11356-022-19056-7 10.1007/s11356-022-19056-7 35320480

[CR51] Haghighi MM, Wright CY, Ayer J, Urban MF, Pham MD, Boeckmann M, Areal A, Wernecke B, Swift CP, Robinson M, Hetem RS, Chersich MF (2021) Climate change and heat-health study group. Impacts of high environmental temperatures on congenital anomalies: a systematic review. Int J Environ Res Public Health 10.3390/ijerph18094910 10.3390/ijerph18094910 PMC8124753 34063033

[CR52] Hahad O, Bayo Jimenez MT, Kuntic M, Frenis K, Steven S, Daiber A, Münzel T. (2022) ‘Cerebral consequences of environmental noise exposure’. Environ Int 10.1016/j.envint.2022.107306 10.1016/j.envint.2022.107306 35635962

[CR53] Hanusch, M. & Fischer, T. B. (2011). SEA and landscape planning. In: Sadler B, Aschemann R, Dusik J, Fischer TB, Partidário M, Verheem R (eds) Handbook of SEA, Earthscan, London, pp 257–173 https://www.taylorfrancis.com/chapters/edit/10.4324/9781849775434-20/sea-landscape-planning-marie-hanusch-thomas-fischer

[CR54] Hernandez Carballo I, Bakola M, Stuckler D (2022) ‘The impact of air pollution on COVID-19 incidence, severity, and mortality: a systematic review of studies in Europe and North America’, Environ Res 215:114155 10.1016/j.envres.2022.114155 10.1016/j.envres.2022.114155 PMC9420033 36030916

[CR55] Hjort M, Mau M, Høj M, Roessler KK (2023) ‘The importance of the outdoor environment for the recovery of psychiatric patients: a scoping review’. Int J Environ Res Public Health 20(3):2240 10.3390/ijerph20032240 10.3390/ijerph20032240 PMC9915437 36767605

[CR56] Hou Y, Zhao Y, Lu J, Wei Q, Zang L, Zhao X (2023) ‘Environmental contamination and health risk assessment of potentially toxic trace metal elements in soils near gold mines—a global meta-analysis’. Environ Pollut 10.1016/j.envpol.2023.121803 10.1016/j.envpol.2023.121803 37187277

[CR57] Huang W, Lin G (2023) ‘The relationship between urban green space and social health of individuals: a scoping review’. Urban For Urban Green 10.1016/j.ufug.2023.127969

[CR58] Janani R, Bhuvana S, Geethalakshmi V, Jeyachitra R, Sathishkumar K, Balu R, Ayyamperumal R (2024) Micro and nano plastics in food: a review on the strategies for identification, isolation, and mitigation through photocatalysis, and health risk assessment. Environ Res 241:11766. 10.1016/j.envres.2023.117666 10.1016/j.envres.2023.117666 37984787

[CR59] Jiao A, Headon K, Han T, Umer W, Wu J (2024) ‘Associations between short-term exposure to wildfire particulate matter and respiratory outcomes: a systematic review’. Sci Total Environ 907 10.1016/j.scitotenv.2023.168134 10.1016/j.scitotenv.2023.168134 39491190

[CR60] Jurgilevich A, Käyhkö J, Räsänen A, Pörsti S, Lagström H, Käyhkö J, Juhola S (2023) ‘Factors influencing vulnerability to climate change-related health impacts in cities—a conceptual framework’. Environ Int 10.1016/j.envint.2023.107837 10.1016/j.envint.2023.107837 36921561

[CR61] Kaur R, Choudhary D, Bali S, Bandral SS, Singh V, Ahmad MA, Rani N, Singh TG, Chandrasekaran B(2024) ‘Pesticides: an alarming detrimental to health and environment’. Sci Total Environ 10.1016/j.scitotenv.2024.170113 10.1016/j.scitotenv.2024.170113 38232846

[CR62] Kharwadkar S, Attanayake V, Duncan J, Navaratne N, Benson J (2022) ‘The impact of climate change on the risk factors for tuberculosis: a systematic review’. Environ Res 10.1016/j.envres.2022.113436 10.1016/j.envres.2022.113436 35550808

[CR63] Kopittke PM, Minasny B, Pendall E, Rumpel C, McKenna BA (2024) ‘Healthy soil for healthy humans and a healthy planet'. Crit Rev Environ Sci Technol 10.1080/10643389.2023.2228651

[CR64] Kristanti RA, Hadibarata T, Syafrudin M, Yılmaz M, Abdullah S (2022) ‘Microbiological contaminants in drinking water: current status and challenges’, *Water, Air, and Soil Pollution*. Springer Science and Business Media Deutschland GmbH 10.1007/s11270-022-05698-3

[CR65] Labib SM, Browning MHEM, Rigolon A, Helbich M, James P (2022) ‘Nature’s contributions in coping with a pandemic in the 21st century: a narrative review of evidence during COVID-19’. Sci Total Environ 10.1016/j.scitotenv.2022.155095 10.1016/j.scitotenv.2022.155095 PMC8983608 35395304

[CR66] Lee GW, Vine K, Atkinson AR, Tong M, Longman J, Barratt A, Bailie R, Vardoulakis S, Matthews V, Rahman KM (2023) ‘Impacts of climate change on health and health services in northern New South Wales, Australia: a rapid review’. Int J Environ Res Public Health 10.3390/ijerph20136285 10.3390/ijerph20136285 PMC10341403 37444133

[CR67] Lee JY, Chia RW, Veerasingam S, Uddin S, Jeon WH, Moon HS, Cha J, Lee J (2024) ‘A comprehensive review of urban microplastic pollution sources, environment and human health impacts, and regulatory efforts’. Sc Total Environ 10.1016/j.scitotenv.2024.174297 10.1016/j.scitotenv.2024.174297 38945237

[CR68] Lévesque-Vézina C, Lapointe M (2024) Health and wellbeing benefits of urban forests in winter: a narrative review. Int J Environ Health Res 35(3):570–584. 10.1080/09603123.2024.2363469 38879884 10.1080/09603123.2024.2363469

[CR69] Li D, Zhang Y, Li X, Zhang K, Lu Y, Brown RD (2023) Climatic and meteorological exposure and mental and behavioral health: a systematic review and meta-analysis. Sci Total Environ 892:164435. 10.1016/j.scitotenv.2023.164435 37257626 10.1016/j.scitotenv.2023.164435 PMC12919713

[CR70] Li H, Browning MHEM, Rigolon A, Larson LR, Taff D, Labib SM, Benfield J, Yuan S, McAnirlin O, Hatami N, Kahn PH (2023) ‘Beyond “bluespace” and “greenspace”: a narrative review of possible health benefits from exposure to other natural landscapes’. Sci Total Environ 10.1016/j.scitotenv.2022.159292 10.1016/j.scitotenv.2022.159292 36208731

[CR71] Liu J, Varghese BM, Hansen A, Borg MA, Zhang Y, Driscoll T, Morgan G, Dear K, Gourley M, Capon A, Bi P (2021) ‘Hot weather as a risk factor for kidney disease outcomes: a systematic review and meta-analysis of epidemiological evidence’. Sci Total Environ 801:149806 10.1016/j.scitotenv.2021.149806 10.1016/j.scitotenv.2021.149806 34467930

[CR72] Liu Q, Wang W, Gu X, Deng F, Wang X, Lin H, Guo X, Wu S (2021) Association between particulate matter air pollution and risk of depression and suicide: a systematic review and meta-analysis. Environ Sci Pollut Res 28:9029–9049 10.1007/s11356-021-12357-3/Published 10.1007/s11356-021-12357-3 33481201

[CR73] Liu Q, Wu Y, Zhao W, Ma J, Qu Y, Chen H, Tian Y, Wu F (2023) ‘Soil environmental criteria in six representative developed countries: soil management targets, and human health and ecological risk assessment’, Crit Rev Environ Sci Technol 10.1080/10643389.2022.2072648

[CR74] Liu Z, Chen X, Cui H, Ma Y, Gao N, Li X, Meng X, Lin H, Abudou H, Guo L, Liu Q (2023) Green space exposure on depression and anxiety outcomes: a meta-analysis. Environ Res 231:116303. 10.1016/j.envres.2023.116303 37268208 10.1016/j.envres.2023.116303

[CR75] Luque-García L, Corrales A, Lertxundi A, Díaz S, Ibarluzea J (2022) ‘Does exposure to greenness improve children’s neuropsychological development and mental health? A Navigation Guide systematic review of observational evidence for associations. Environ Res 10.1016/j.envres.2021.112599 10.1016/j.envres.2021.112599 34932982

[CR76] Markozannes G, Pantavou K, Rizos EC, Sindosi O, Tagkas C, Seyfried M, Saldanha IJ, Hatzianastassiou N, Nikolopoulos GK, Ntzani E (2022) Outdoor air quality and human health: an overview of reviews of observational studies. Environ Pollut 306:119309. 10.1016/j.envpol.2022.119309 35469927 10.1016/j.envpol.2022.119309

[CR77] Marselle MR, Hartig T, Cox DTC, de Bell S, Knapp S, Lindley S, Triguero-Mas M, Böhning-Gaese K, Braubach M, Cook PA, de Vries S, Heintz-Buschart A, Hofmann M, Irvine KN, Kabisch N, Kolek F, Kraemer R, Markevych I, Martens D, Müller R, Nieuwenhuijsen M, Potts JM, Stadler J, Walton S, Warber SL, Bonn A (2021) Pathways linking biodiversity to human health: a conceptual framework. Environ Int 150:106420. 10.1016/j.envint.2021.106420 33556912 10.1016/j.envint.2021.106420

[CR78] Marselle MR, Lindley SJ, Cook PA, Bonn Aletta (2021) ‘Biodiversity and health in the urban environment. Curr Environ Health Rep 8:146–156. 10.1007/s40572-021-00313-9/Published 33982150 10.1007/s40572-021-00313-9 PMC8115992

[CR79] Matthews K, Cavagnaro T, Weinstein P, Stanhope J (2024) ‘Health by design; optimising our urban environmental microbiomes for human health’. Environ Res 10.1016/j.envres.2024.119226 10.1016/j.envres.2024.119226 38797467

[CR80] Menne B, Aragon De Leon E, Bekker M, Mirzikashvili N, Morton S, Shriwise A, Wippel C (2020) Health and well-being for all: an approach to accelerating progress to achieve the Sustainable Development Goals (SDGs) in countries in the WHO European Region. Eur J Public Health 30:I3–I9. 10.1093/eurpub/ckaa026 32391901 10.1093/eurpub/ckaa026 PMC7213559

[CR81] Moeller, D.W. (2005) Environmental health. 3rd edn. Cambridge, Mass, Harvard University Press

[CR82] Moon J (2021) The effect of the heatwave on the morbidity and mortality of diabetes patients; a meta-analysis for the era of the climate crisis. Environ Res 195:110762. 10.1016/j.envres.2021.110762 33515577 10.1016/j.envres.2021.110762

[CR83] Mygind L, Kurtzhals M, Nowell C, Melby PS, Stevenson MP, Nieuwenhuijsen M, Lum JAG, Flensborg-Madsen T, Bentsen P, Enticott PG (2021) ‘Landscapes of becoming social: a systematic review of evidence for associations and pathways between interactions with nature and socioemotional development in children’. Environ Int 10.1016/j.envint.2020.106238 10.1016/j.envint.2020.106238 33189991

[CR84] Nath S, Enerijiofi KE, Astapati AD, Guha A (2024) Microplastics and nanoplastics in soil: Sources, impacts, and solutions for soil health and environmental sustainability. J Environ Qual 53:1048–1072. 10.1002/jeq2.20625 39246015 10.1002/jeq2.20625

[CR85] Natur S, Damri O, Agam G (2022) The effect of global warming on complex disorders (mental disorders, primary hypertension, and type 2 diabetes). Int J Environ Res Public Health 19(15):9398. 10.3390/ijerph19159398 35954764 10.3390/ijerph19159398 PMC9368177

[CR86] Nishi M, Hashimoto S (2022) ‘Health and landscape approaches: a comparative review of integrated approaches to health and landscape management’. Environ Sci Policy 10.1016/j.envsci.2022.06.015

[CR87] Nuruzzaman M, Bahar MM, Naidu R (2025) ‘Diffuse soil pollution from agriculture: Impacts and remediation’. Sci Total Environ 10.1016/j.scitotenv.2025.178398 10.1016/j.scitotenv.2025.178398 39808904

[CR88] Patwary MM, Bardhan M, Haque MA, Moniruzzaman S, Gustavsson J, Khan MMH, Koivisto J, Salwa M, Mashreky SR, Rahman AKMF, Tasnim A, Islam MR, Alam MA, Hasan M, Harun MAYA, Nyberg L, Islam MA (2024) ‘Impact of extreme weather events on mental health in South and Southeast Asia: a two decades of systematic review of observational studies’. Environ Res 10.1016/j.envres.2024.118436 10.1016/j.envres.2024.118436 38354890

[CR89] Picetti R, Deeney M, Pastorino S, Miller MR, Shah A, Leon DA, Dangour AD, Green R (2022) ‘Nitrate and nitrite contamination in drinking water and cancer risk: a systematic review with meta-analysis’. Environ Res 10.1016/j.envres.2022.112988 10.1016/j.envres.2022.112988 35217009

[CR90] Potter JD, Brooks C, Donovan G, Cunningham C, Douwes J (2023) ‘A perspective on green, blue, and grey spaces, biodiversity, microbiota, and human health’. Sci Total Environ 10.1016/j.scitotenv.2023.164772 10.1016/j.scitotenv.2023.164772 37308017

[CR91] Pritchett N, Spangler EC, Gray GM, Livinski AA, Sampson JN, Dawsey SM, Jones RR (2022) Exposure to outdoor particulate matter air pollution and risk of gastrointestinal cancers in adults: a systematic review and meta-analysis of epidemiologic evidence. Environ Health Perspect 130(3):36001. 10.1289/EHP9620 35234536 10.1289/EHP9620 PMC8890324

[CR92] Pyper R, Cave B, Purdy J, McAvoy H (2021) Health impact assessment guidance: a manual, standalone health impact assessment and health in environmental assessment. Dublin and Belfast 10.14655/11971-1084894

[CR93] Pyper R, Fischer TB, Muthoora T, Cave B (2022) Learning from practice: case studies of health in strategic environmental assessment and environmental impact assessment across the WHO European Region. Copenhagen https://apps.who.int/iris/handle/10665/353810?show=ful

[CR94] Rasking L, Vanbrabant K, Bové H, Plusquin M, De Vusser K, Roels HA, Nawrot TS (2022) Adverse Effects of fine particulate matter on human kidney functioning: a systematic review. Environ Health Glob Access Sci Source 21(1):24. 10.1186/s12940-021-00827-7 10.1186/s12940-021-00827-7 PMC8822715 35135544

[CR95] Reyes-Riveros R, Altamirano A, De La Barrera F, Rozas-Vásquez D, Vieli L, Meli P (2021) ‘Linking public urban green spaces and human well-being: a systematic review’. Urban For Urban Green 10.1016/j.ufug.2021.127105

[CR96] Robinson JM, Breed AC, Camargo A, Redvers N, Breed MF (2024) ‘Biodiversity and human health: a scoping review and examples of underrepresented linkages’. Environ Res 10.1016/j.envres.2024.118115 10.1016/j.envres.2024.118115 38199470

[CR97] Roue Le Gall A, Le Gall J, Potelin J, Cuzin Y (2014) Agir pour un urbanisme favorable à la santé, concepts & outils. Guide

[CR98] Roue Le Gall A, van Gastel B, Dardier G, Legea M (2024) HIA guidelines in georgia: practical application of health in environmental assessment. https://www.expertisefrance.fr/documents/20182/861856/Health+Impact+Assessment+Guidelines+in+Georgia/4c918cec-e035-3032-ab18-e670af9a85b8

[CR99] Salvo G, Lashewicz BM, Doyle-Baker PK, McCormack GR (2018) Neighbourhood built environment influences on physical activity among adults: a systematized review of qualitative evidence. Int J Environ Res Public Health 15(5):897. 10.3390/ijerph15050897 29724048 10.3390/ijerph15050897 PMC5981936

[CR100] Sardar MF, Younas F, Li H, Ali J, Zhu P, Yu X, Cui Z, Guo W (2025) ‘Current scenario of emerging pollutants in farmlands and water reservoirs: prospects and challenges’. Ecotoxicol Environ Safe 10.1016/j.ecoenv.2025.117829 10.1016/j.ecoenv.2025.117829 39908865

[CR101] Scottish Environment Protection Agency (2019) Guidance on consideration of human health in Strategic Environmental Assessment - Strategic Environmental Assessment SEPA Guidance Note 5 - Version 3 https://www.gov.scot/policies/environmental-assessment/strategic-environmental-assessment-sea/

[CR102] Seidler A, Schubert M, Mehrjerdian Y, Krapf K, Popp C, van Kamp I, Ögren M, Hegewald J (2023) ‘Health effects of railway-induced vibration combined with railway noise—a systematic review with exposure-effect curves’, Environ Res 10.1016/j.envres.2023.116480 10.1016/j.envres.2023.116480 37352957

[CR103] Setu S, Strezov V (2025) ‘Impacts of non-ferrous metal mining on soil heavy metal pollution and risk assessment’. Sci Total Environ 10.1016/j.scitotenv.2025.178962 10.1016/j.scitotenv.2025.178962 40022981

[CR104] Shi W, Wu N, Zhang Z, Liu Y, Chen J, Li J (2024) ‘A global review on the abundance and threats of microplastics in soils to terrestrial ecosystem and human health’. Sci Total Environ 10.1016/j.scitotenv.2023.169469 10.1016/j.scitotenv.2023.169469 38154650

[CR105] Singh VK, Singh P, Karmakar M, Leta J, Mayr P (2021) The journal coverage of web of science, scopus and dimensions: a comparative analysis. Scientometrics 126(6):5113–5142. 10.1007/s11192-021-03948-5

[CR106] Smith MG, Cordoza M, Basner M (2022) ‘Environmental noise and effects on sleep: an update to the WHO systematic review and meta-analysis’. Environ Health Perspect 10.1289/EHP10197 10.1289/EHP10197 PMC9272916 35857401

[CR107] Song S, Gao Z, Zhang X, Zhao X, Chang H, Zhang J, Yu Z, Huang C, Zhang H (2023) Ambient fine particulate matter and pregnancy outcomes: an umbrella review. Environ Res 235:116652. 10.1016/j.envres.2023.116652 37451569 10.1016/j.envres.2023.116652

[CR108] Sun M, Li T, Sun Q, Ren X, Sun Z, Duan J (2023) Associations of long-term particulate matter exposure with cardiometabolic diseases: a systematic review and meta-analysis. Sci Total Environ 903:166010. 10.1016/j.scitotenv.2023.166010 37541522 10.1016/j.scitotenv.2023.166010

[CR109] Syafrudin M, Kristanti RA, Yuniarto A, Hadibarata T, Rhee J, Al-Onazi WA, Algarni TS, Almarri AH, Al-Mohaimeed AM (2021) ‘Pesticides in drinking water-a review’. Int J Environ Res Public Health 10.3390/ijerph18020468 10.3390/ijerph18020468 PMC7826868 33430077

[CR110] Syed S, O’Sullivan TL, Phillips KP (2022) Extreme heat and pregnancy outcomes: a scoping review of the epidemiological evidence. Int J Environ Res Public Health 19(4):2412. 10.3390/ijerph19042412 35206601 10.3390/ijerph19042412 PMC8874707

[CR111] Thompson R, Smith RB, Bou Karim Y, Shen C, Drummond K, Teng C, Toledano MB (2022) ‘Noise pollution and human cognition: an updated systematic review and meta-analysis of recent evidence’. Environ Int 10.1016/j.envint.2021.106905 10.1016/j.envint.2021.106905 34649047

[CR112] Thompson R, Smith RB, Karim YB, Shen C, Drummond K, Teng C, Toledano MB (2023) Air pollution and human cognition: a systematic review and meta-analysis. Sci Total Environ 859:160234. 10.1016/j.scitotenv.2022.160234 36427724 10.1016/j.scitotenv.2022.160234

[CR113] UK Health Security Agency (2023) Noise pollution: mapping the health impacts of transportation noise in England https://ukhsa.blog.gov.uk/2023/06/29/noise-pollution-mapping-the-health-impacts-of-transportation-noise-in-england/

[CR114] UN - United Nations (2015) Transforming Our World: The 2030 Agenda for Sustainable Development. sustainabledevelopment.un.org

[CR115] UNECE - United Nations Economic Commission for Europe (2003) UNECE Protocol on Strategic Environmental Assessment to the Convention on Environmental Impact Assessment in a Transboundary Context https://unece.org/text-protocol

[CR116] UNECE - United Nations Economic Commission for Europe (2022). Health in Strategic Environmental Assessment https://unece.org/environmental-policy/events/second-sub-regional-workshop-practical-application-strategic

[CR117] Velazquez-Meza ME, Galarde-López M, Carrillo-Quiróz B, Alpuche-Aranda CM (2022) ‘Antimicrobial resistance: One Health approach’. Veterinary World, Veterinary World, pp. 743–749 10.14202/vetworld.2022.743-749 10.14202/vetworld.2022.743-749 PMC9047147 35497962

[CR118] Verscheure P, Honnay O, Speybroeck N, Daelemans R, Bruffaerts N, Devleesschauwer B, Ceulemans T, Van Gerven L, Aerts R, Schrijvers R (2023) ‘Impact of environmental nitrogen pollution on pollen allergy: a scoping review’. Sci Total Environ 893:164801. 10.1016/j.scitotenv.2023.164801 37321510 10.1016/j.scitotenv.2023.164801

[CR119] Vienneau D, Wicki B, Flückiger B, Schäffer B, Wunderli JM, Röösli M (2024) ‘Long-term exposure to transportation noise and diabetes mellitus mortality: a national cohort study and updated meta-analysis’. Environ Health A Global Access Sci Source 23(1) 10.1186/s12940-024-01084-0 10.1186/s12940-024-01084-0 PMC11068573 38702725

[CR120] Weeda LJZ, Bradshaw CJA, Judge MA, Saraswati CM, Le Souëf PN (2024) ‘How climate change degrades child health: a systematic review and meta-analysis’. Sci Total Environ 10.1016/j.scitotenv.2024.170944 10.1016/j.scitotenv.2024.170944 38360325

[CR121] Williams C, Fisher P (2007) The UK’s draft guidance on health in strategic environmental assessment - consultation document https://healthimpactassessment.pbworks.com/f/Draft+guidance+on+health+in+SEA+-+DH+England+-+2007.pdf

[CR122] Winkler M, Viliani F, Knoblauch A, Cave B, Divall M, Ramesh G, HarrisRoxas B, Furu P (2021) International best practice principles: health impact assessment (2nd edition); International Association for Impact Assessment, Fargo, ND

[CR123] World Bank (2022) Putting pandemics behind us: investing in one health to reduce risks of emerging infectious diseases. Washington, DC http://hdl.handle.net/10986/38200

[CR124] WHO - World Health Organization (2018) Environmental Noise Guidelines for the European Region. Geneva https://www.who.int/europe/publications/i/item/9789289053563

[CR125] WHO - World Health Organization (2020) Constitution of the World Health Organization - Basic Documents: forty-ninth edition (including amendments adopted up to 31 May 2019). Geneva: World Health Organization https://www.who.int/about/governance/constitution

[CR126] WHO - World Health Organization (2021) WHO global air quality guidelines. Geneva https://www.who.int/publications/i/item/9789240034228

[CR127] WHO - World Health Organization (2023) One Health, World Health Organization https://www.who.int/news-room/fact-sheets/detail/one-health

[CR128] WHO - World Health Organization (2024) Determinants of health https://www.who.int/news-room/questions-and-answers/item/determinants-of-health

[CR129] WHO - World Health Organization (2025) Water, sanitation and hygiene (WASH) https://www.who.int/health-topics/water-sanitation-and-hygiene-wash#tab=tab_1

[CR130] Wyer KE, Kelleghan DB, Blanes-Vidal V, Schauberger G, Curran TP (2022) Ammonia emissions from agriculture and their contribution to fine particulate matter: a review of implications for human health’. J Environ Manag 323:116285. 10.1016/j.jenvman.2022.116285 10.1016/j.jenvman.2022.116285 36261990

[CR131] Zhang H, Chen Y, Wang J, Wang Y, Wang L, Duan Z (2022) ‘Effects of temperature on the toxicity of waterborne nanoparticles under global warming: facts and mechanisms’. Mar Environ Res 181:105757. 10.1016/j.marenvres.2022.105757 36208504 10.1016/j.marenvres.2022.105757

[CR132] Zhang M, Wang C, Zhang X, Song H, Li Y (2022) ‘Association between exposure to air pollutants and attention-deficit hyperactivity disorder (ADHD) in children: a systematic review and meta-analysis’. Int J Environ Health Res 32(1):207–219. 10.1080/09603123.2020.1745764 32248699 10.1080/09603123.2020.1745764

[CR133] Zhang P, Yang M, Lan J, Huang Y, Zhang J, Huang S, Yang Y, Ru J (2023) ‘Water quality degradation due to heavy metal contamination: health impacts and eco-friendly approaches for heavy metal remediation’. Toxics 10.3390/toxics11100828 10.3390/toxics11100828 PMC10611083 37888679

[CR134] Zhang R, Zhang CQ, Rhodes RE (2021) ‘The pathways linking objectively-measured greenspace exposure and mental health: a systematic review of observational studies’. Environ Res 10.1016/j.envres.2021.111233 10.1016/j.envres.2021.111233 33933490

[CR135] Zhao Y, Bao WW, Yang BY, Liang JH, Gui ZH, Huang S, Chen YC, Dong GH, Chen YJ (2022) ‘Association between greenspace and blood pressure: a systematic review and meta-analysis’. Sci Total Environ 817:152513. 10.1016/j.scitotenv.2021.152513 35016929 10.1016/j.scitotenv.2021.152513

[CR136] Zheng J, Zhang H, Shi J, Li X, Zhang J, Zhang K, Gao Y, He J, Dai J, Wang J (2024) Association of air pollution exposure with overweight or obesity in children and adolescents: a systematic review and meta–analysis’. Sci Total Environ 910:168589. 10.1016/j.scitotenv.2023.168589 37984657 10.1016/j.scitotenv.2023.168589

[CR137] Zhu J, Chen J, Wang K, Yan H, Liu Q, Lan Y, Ren L, Wu S (2024) ‘Exposure to ambient black carbon and particulate matter during pregnancy in associations with risk of pre-eclampsia: a meta-analysis based on population-based studies’. Environ Pollut 343 10.1016/j.envpol.2023.123230 10.1016/j.envpol.2023.123230 38158011

[CR138] Zhu R, Yuan L, Pan Y, Wang Y, Xiu D, Liu W (2024) ‘Effects of natural sound exposure on health recovery: a systematic review and meta-analysis’. Sci Total Environ 10.1016/j.scitotenv.2024.171052 10.1016/j.scitotenv.2024.171052 38373459

[CR139] Zhu Z, Ji B, Tian J Yin P (2025) ‘Heat exposure and respiratory diseases health outcomes: an umbrella review’. Sci Total Environ 10.1016/j.scitotenv.2025.179052 10.1016/j.scitotenv.2025.179052 40056553

